# Survival analysis of adult patients with ALL in Mexico City: first report from the Acute Leukemia Workgroup (ALWG) (GTLA)

**DOI:** 10.1002/cam4.1513

**Published:** 2018-05-07

**Authors:** Erick Crespo‐Solis, Karla Espinosa‐Bautista, Martha Alvarado‐Ibarra, Etta Rozen‐Fuller, Fernando Pérez‐Rocha, Chantal Nava‐Gómez, Maricela Ortiz‐Zepeda, José Luis Álvarez‐Vera, Christian Omar Ramos‐Peñafiel, Luis Antonio Meillón‐García, Sergio Rodríguez‐Rodríguez, Alan Pomerantz‐Okon, Francisco Javier Turrubiates‐Hernández, Roberta Demichelis‐Gómez

**Affiliations:** ^1^ Hospital Regional de Alta Especialidad de Ciudad Victoria Ciudad Victoria Tamaulipas México; ^2^ Instituto Nacional de Cancerología Ciudad de México México; ^3^ Centro Médico Nacional 20 de Noviembre ISSSTE Ciudad de México México; ^4^ Hospital General de México Ciudad de México México; ^5^ Centro Médico Nacional Siglo XXI IMSS Ciudad de México México; ^6^ Instituto Nacional de Ciencias Médicas y Nutrición Salvador Zubirán Ciudad de México México

**Keywords:** Acute lymphoblastic leukemia, AYA, GTLA, survival

## Abstract

Acute lymphoblastic leukemia (ALL) is a hematologic malignancy characterized by the clonal expansion of hematopoietic lymphoid progenitors. With new target therapies, the survival of adults with ALL has improved in the past few decades. Unfortunately, there are no large ALL patient series in many Latin American countries. Data from the Acute Leukemia Workgroup that includes five Mexico City referral centers were used. Survival was estimated for adult patients with ALL during 2009–2015. In total, 559 adults with ALL were included. The median age was 28 years; 67% were classified into the adolescent and young adult group. Cytogenetic information was available in 54.5% of cases. Of the 305 analyzed cases, most had a normal caryotype (70.5%) and Philadelphia‐positive was present in 16.7%. The most commonly used treatment regimen was hyper‐CVAD. In approximately 20% of cases, there was considerable delay in the administration of chemotherapy. Primarily refractory cases accounted for 13.1% of patients. At the time of analysis, 26.7% of cases had survived. The 3‐year overall survival was 22.1%. The main cause of death was disease progression in 228 (55.6%). Clinical and public health strategies are needed to improve diagnosis, treatment and survivorship care for adult with ALL. This multicentric report represents the largest series in Mexico of adult ALL patients in which a survival analysis and risk identification were obtained.

## Introduction

Acute lymphoblastic leukemia (ALL) is a hematologic malignancy characterized by the clonal expansion of hematopoietic progenitors committed to the lymphoid lineage (lymphoblasts). These cells progressively replace hematopoietic tissue and may lead to decreases of all three cell lines 1. In accordance with the 2016 WHO criteria, ALL is classified in 13 categories based on the cellular immunophenotype and genomic abnormalities 2.

Prognostic factors have changed over time due to the generation of risk‐adapted treatment regimens, including hematopoietic stem cell transplant (HSCT), as well as drugs such as rituximab, imatinib, and dasatinib directed against surface antigens and molecular targets, and more recently, blinatumomab and inotuzumab appear to be promising drugs in terms of patient survival 3, 4, 5, 6, 7. In general, currently accepted high‐risk factors are T‐cell precursor leukemias, age, leukocytosis, and genetic factors 8, 9, 10, 11, 12, 13, 14.

As a result of these advances, the survival of adults with ALL has improved in the past few decades. In developed societies, complete remission (CR) rates range between 85% and 90%; however, long‐term overall survival (OS) rates remain between 40% and 50% 13, 14, 15, 16, particularly as a result of relapse and disease progression. The MD Anderson Cancer Center (MDACC) group has reported CR rates above 90% and OS of 60% at 5 years in adolescent and young adult (AYA) ALL patients treated with hyper‐CVAD and the *Augmented Berlin–Frankfurt–Münster* (ABFM) protocol 17.

Unfortunately, the course of adult patients in Latin America yields less favorable results 18, 19. A study conducted in Brazil reported CR >90% and 5‐year OS curves of 35% with the hyper‐CVAD regimen 20. Unfortunately, there are no large ALL patient series in many Latin American countries.

Reports in Mexico have referred CR rates between 60% and 80% with hyper‐CVAD and other locally designed institutional regimens. Among the limitations of studies published in Mexico are the use of diverse treatment protocols that make interinstitutional comparisons difficult, the series are small, there are deficiencies in cytogenetic results (inaccessible or technique failures), all impinging on the reported incidence of Philadelphia‐positive ALL (Ph^+^‐ALL); also, most studies lack a survival analysis and risk factor identification 1, 21, 22, 23, 24. A recent series of 94 patients at the *Hospital Universitario de Monterrey, Nuevo León* 25 treated with the *Berlin–Frankfurt–Münster* (BFM) regimen, reported a CR of 71.3% and a 5‐year OS of 31.1%. This study did include a survival analysis and the identification of risk factors but patient follow‐up was relatively short (34 months).

The aim of this study was to conduct a survival analysis in a large series of adult ALL patients in Mexico City referral centers.

## Material and Methods

The Acute Leukemia Workgroup (GTLA, by its initials in Spanish) was the result of an initiative of the *Agrupación Mexicana para el Estudio de la Hematología, A.C. (*AMEH*)* to promote research in acute leukemia in Mexico. This is a retrospective and multicentric study of adult patients with ALL, between 2009 and 2015, and the first report presented by the GTLA.

### ALL diagnosis and classification

The patient charts of individuals fulfilling the WHO ALL diagnostic criteria were selected. Demographic data, laboratory results, bone marrow (BM) aspirate, BM biopsy, immunophenotype or immunohistochemistry results (if the immunophenotype was unavailable) were collected. Likewise, all patients with a Ph^+^ determined by conventional caryotype or fluorescent in situ hybridization (FISH), were also included in the registry.

### Treatment and response to treatment

We recorded the treatment regimens and they were classified, in general, into previously published classic treatment protocols, that is, hyper‐CVAD or BFM 26, 27, institutional regimens, and pediatric regimens. Patients who underwent HSCT and those who received imatinib or dasatinib were also registered.

To define the response to treatment, we estimated the CR, relapse, OS, and disease‐free survival (DFS). CR was established in accordance with the Cheson criteria that denote the absence of extramedullary leukemia, the lack of peripheral blood blasts, a BM blast percentage below 5%, a neutrophil count ≥1.5 × 10^9^/L, and platelets ≥100 × 10^9^/L 28. Disease relapse referred to those cases in which after achieving CR, there was new evidence of disease documented by ≥5% blasts in BM, extramedullary leukemia at any site or clear evidence of leukemia in peripheral blood (PB). DFS was defined as the time lapse between CR and the first relapse or the last day of follow‐up. OS referred to the time lapse between diagnosis until the patient's death or the last day of follow‐up.

Treatment delay refers to instances in which administration of the chemotherapy protocol was over 30% of the expected time required to complete the regimen. Induction treatment‐related mortality was defined as deaths that occurred within 30 days after administering the induction regimen, during the postchemotherapy myelosuppression period and that had no associated manifestations of refractory disease or clear disease progression. To analyze the response by age groups, we created the following classification: AYAs ≤39 years; elderly adults ≥60 years, and adults between 40 and 59 years.

### Statistical analysis

Continuous variables were described as medians and intervals, and categorical variables as frequencies and proportions. OS and DFS were analyzed with the Kaplan–Meier method. Proportion differences between groups were compared with the chi‐square test or Fisher's exact test for parametric and nonparametric distributions, respectively. Between‐group numerical variable distribution was analyzed with Student's *t*‐test and Mann–Whitney U test, for parametric and nonparametric distributions, respectively. Cox proportional hazards analysis (uni‐ and multivariate) was used to determine possible risk factors relating to OS and DFS. The variables fulfilling each of the following requirements were chosen for the multivariate analysis:

1. Representativity (available data with less than 20% losses)
2. That made biological sense
3. Hazards ratio ≥1.5
4. No important correlation with another biologically related variable, that is, leukocytes and lactate dehydrogenase (LDH)
5. *P* value <0.05

### Ethical aspects

The study protocol was approved by the institutional Ethics Committee of every participating center; a signed consent form was not required since the study's design is retrospective. The study's registration number is: *ClinicalTrials.gov Identifier NCT02990104*.

### Declarations

The AMEH was financially sponsored by Applied Molecular Genetics Inc. (AMGEN) laboratories to conduct the study.

## Results

A total of (559) adult patients with ALL and treated between 2009 and 2015 in five referral centers in Mexico City were included in the study. The centers were Instituto Nacional de Cancerología (INCAN), Instituto Nacional de Ciencias Médicas y Nutrición Salvador Zubirán (INCMNSZ), Hospital General de México (HGM), Centro Médico Nacional 20 de Noviembre ISSSTE (CMN 20 Nov), and Centro Médico Nacional Siglo XXI IMSS (CMN SXXI).

Of the 559 patients, 258 (46.2%) were females and 301 (53.8%) were males. Their median age was 28 years (interval, 14–81 years). We must highlight the fact that 376 cases (67.3%) were classified into the AYA group, 138 cases were adults (24.7%) and 45 cases (8.1%) were elderly adults. Tumor lysis syndrome (TLS) was detected in 9.8% of patients, and abnormal liver function tests with values 2.5‐fold above the normal upper limit (LFT ≥2.5 ULN) were found in 66 of the 494 (13.4%) cases. In terms of physical performance status, an ECOG of 0–1 was obtained in 68.5% of cases. Based on the immunophenotype, the disease was adequately subclassified in 509 (91%) of patients, and most cases (87.4%) were pre‐B ALL. Cytogenetic information was available in 54.5% of cases; missing data were either due to unavailability or lack of growth in metaphase. Of the 305 analyzed cases, most had a normal caryotype (70.5%) and Ph^+^ was present in 16.7%. In the AYA group, there were 77.8% cases with a normal caryotype and 10.8% were Ph^+^‐ALL; these differences were statistically significant (*P = *0.0001 in both cases). It is important to point out that FISH could be done in 290 patients in order to detect the Philadelphia chromosome. Only 15.5% were positive by this method. Polymerase chain reaction (PCR) studies were not performed.

Patients were considered high risk if they had any of the following features at diagnosis: leukocyte count ≥30 × 10^9^/L in B‐cell lineage cases; ≥100 × 10^9^/L in T‐cell lineage cases and poor prognostic cytogenetics; or the absence of an early CR. With these characteristics, 52.1% of patients fulfilled high‐risk criteria (Table 1).

**Table 1:** Demographic, clinical, and laboratory characteristics of patients with ALL

	Entire cohort *N* = 559	AYAs *N* = 376	Adults and elderly adults *N* = 183
Institution, *n* (%)			
INCAN	158 (28.2)	114 (30.3)	44 (24)
INCMNSZ	156 (28)	94 (25)	62 (33.9)
HGM	119 (21.2)	90 (23.9)	29 (15.8)
CMN 20 Nov	78 (14)	49 (13)	29 (15.8)
CMN SXXI	48 (8.5)	29 (7.7)	19 (10.4)
Gender, *n* (%)			
Female	258 (46.2)	179 (47.6)	79 (43.2)
Male	301 (53.8)	197 (52.4)	104 (56.8)
Age, median (range)	28 (14–81)	22 (14–39)	52 (40–81)
Evaluated obesity, *n* (%)	339 (60.6)	223 (59.3)	116 (63.4)
Present	63 (18.5)	39 (17.4)	24 (20.6)
ECOG, *n* (%)	504 (90.2)	331 (88)	173 (94.5)
0–1	345 (68.4)	240 (72.5)	105 (60.6)
2	127 (25.2)	70 (21.2)	57 (33)
3	31 (6.2)	20 (6)	11 (6.3)
4	1 (0.2)	1 (0.3)	0 (0)
TLS, *n* (%)	55 (9.8)	38 (10.1)	17 (9.3)
Hyperleukocytosis syndrome, *n* (%)	75 (13.4)	45 (12)	30 (16.4)
Evaluated LFT ≥2.5 ULN, *n* (%)	494 (88.4)	332 (88.3)	162 (88.5)
Abnormality	66 (13.4)	45 (13.6)	21 (13)
Evaluated Immunophenotype, *n* (%)	509 (91)	333 (88.6)	176 (96.2)
Precursors B	445 (87.4)	292 (87.6)	153 (87)
Mature B	49 (9.6)	32 (9.6)	17 (9.6)
Precursors T	15 (2.9)	9 (2.8)	6 (3.4)
Evaluated CD20, *n* (%)	525 (94)	352 (94)	173 (94.5)
Positive	252 (48)	164 (46.5)	88 (50.8)
Evaluated CD34, *n* (%)	508 (91)	338 (90)	170 (92.8)
Positive	397 (78.1)	260 (77)	137 (80.5)
Evaluated aberrant myeloid, *n* (%)	429 (76.7)	275 (73)	154 (84.2)
Present	111 (25.8)	75 (27.2)	36 (23.3)
Available cytogenetics, *n* (%)	305 (55)	203 (53.4)	102 (55.7)
Normal caryotype	215 (70.5)	158 (77.8)^a^	57 (55.8)
Ph^+^	51 (16.7)	22 (10.8)^a^	29 (28.4)
Hypodiploid	6 (2)	2 (1)	4 (4)
Hyperdiploid	10 (3.3)	7 (3.4)	3 (3)
Others	23 (7.5)	14 (7)	9 (8.8)
High risk, *n* (%)	291 (52.1)	182 (48.4)	109 (59.5)
Median (interval)			
Hemoglobin, g/dL	8.1 (2.2–16.8)	8 (2.2–16.8)	8.3 (3.3–16.4)
Leukocytes, ×10^9^/L	9.25 (0.1–690)	10.1 (0.3–550.9)	7.7 (0.1–690)
Platelets, ×10^9^/L	36 (1–446)	36.5 (1–446)	34 (4–373)
Blasts, %			
PB	9 (0–100)	8 (0–100)	12 (0–95)
BM	84 (0–100)	85 (0–100)	83 (2–100)
Creatinine, mg/dL	0.8 (0.2–9.4)	0.79 (0.3–9.4)	0.8 (0.2–3.4)
Uric acid, mg/dL	5.3 (0.7–28)	5.6 (1.4–28)	4.75 (0.7–20.3)
LDH, U/L	406 (79–8541)	391 (79–8541)	417.5 (86–5950)
Glucose, mg/dL	105 (38–487)	100.5 (38–487)	112 (62–483)
Treatment regimen, *n* (%)			
hyper‐CVAD	263 (47)	184 (49)	79 (43.2)
Institutional	202 (36.1)	148 (39.3)	54 (29.5)
Pediatric	57 (10.2)	28 (7.4)	29 (15.8)
Others	7 (1.3)	5 (1.3)	1 (0.5)
Patients with treatment protocol administration delay, *n* (%)	105 (19.1)	66 (17.7)	39 (21.8)
Imatinib, *n* (%)	25 (4.5)	15 (4)	10 (5.5)
Dasatinib, *n* (%)	19 (3.4)	5 (1.3)	14 (7.7)
Allogenic HSCT, *n* (%)	32 (5.7)	25 (6.6)	7 (3.8)

a *P *= 0.0001.

The most commonly used treatment regimen was hyper‐CVAD (47% of cases), followed by institutional protocols in 36.1% cases and pediatric regimens in 10.2%. In approximately 20% of cases, there was considerable delay in the administration of chemotherapy. Imatinib and dasatinib were administered to 4.5% and 3.4% of cases, respectively. These drugs were administered to Ph^+^‐ALL patients as follows: imatinib was administered to 17 patients (34%), dasatinib to 18 (35%), and 16 patients (31%) received no TKI. Early CR was obtained in 387 of the 559 (69.2%) cases, and 16.3% required a second chemotherapy cycle to achieve CR, yielding an overall CR rate of 75.3%. Primarily refractory cases accounted for 13.1% of patients. Treatment induction‐related mortality was 10.6%, while another 10.6% of patients died in CR during other treatment stages; hence, the mortality rate related to chemotherapy throughout treatment was 21.2% (Table 2).

**Table 2:** Treatment response and mortality

	Entire cohort *N* = 559	AYAs *N* = 376	Adults and elderly adults *N* = 183
Early CR, *n* (%)	387 (69.2)	275 (73.1)	112 (61.2)
Reinduction^a^, *n* (%)	91 (16.3)	66 (17.6)	25 (13.7)
CR, *n* (%)	421 (75.3)	301 (80.1)	120 (65.6)
Refractory, *n* (%)	73 (13.1)	50 (13.3)	23 (12.6)
Mortality during induction, *n* (%)	59 (10.6)	26 (6.9)	33 (18)
Mortality in CR, *n* (%)	59 (10.6)	39 (10.4)	20 (10.9)
Relapse^b^, *n* (%)	264/421 (62.7)	186/301 (61.7)	78/120 (65)
CNS^c^	34 (12.8)	27 (14.5)	7 (9)
BM	201 (76.1)	137 (74)	64 (82)
CNS and BM	14 (5.3)	11 (6)	3 (3.8)
Others	16 (6)	10 (5.3)	4 (5.1)
Death, *n* (%)	410 (73.3)	267 (71)	143 (78.1)
Infection	110 (26.8)	66 (24.7)	44 (30.8)
Hemorrhage	34 (8.2)	25 (9.3)	9 (6.3)
Toxicity	11 (2.6)	7 (2.6)	4 (2.7)
Disease progression	228 (55.6)	152 (56.9)	76 (53.1)
Unrelated	5 (1.2)	1 (0.4)	4 (2.7)
Unknown	23 (5.6)	18 (6.7)	5 (3.5)
Median (CI 95%)			
DFS, months	16.0 (13.30–18.69)	16.9 (13.69–20.27)	13.4 (11.23–15.56)
OS, months	12.9 (11.49–14.46)	14.1 (11.76–16.48)^d^	10.5 (8.28–12.87)^d^

a Proportion of patients requiring a second dose of the induction regimen to achieve CR.

b Estimated from the CR cases.

c Central nervous system.

d *P *= 0.0001.

Thirty‐two patients (5.7%) were managed with an allogenic HSCT; from these 32 patients, 21 (65.6%) have died. The main causes of death were disease progression (47.6%) and infection (38%).

At the time of analysis, 26.7% of cases had survived, with a median OS of 12.97 months (CI 95%; 11.49–14.46 months) and a DFS of 16 months (CI 95%; 13.30–18.69 months); the OS of patients that achieved CR was 17 months (CI 95%; 15.14–18.85 months). The 3‐year OS was 22.1%, and by age group it was distributed as follows: AYAs (25.7%), adults (17.4%), and elderly adults (0%) (*P *=* *0.0001).

**Figure 1:**
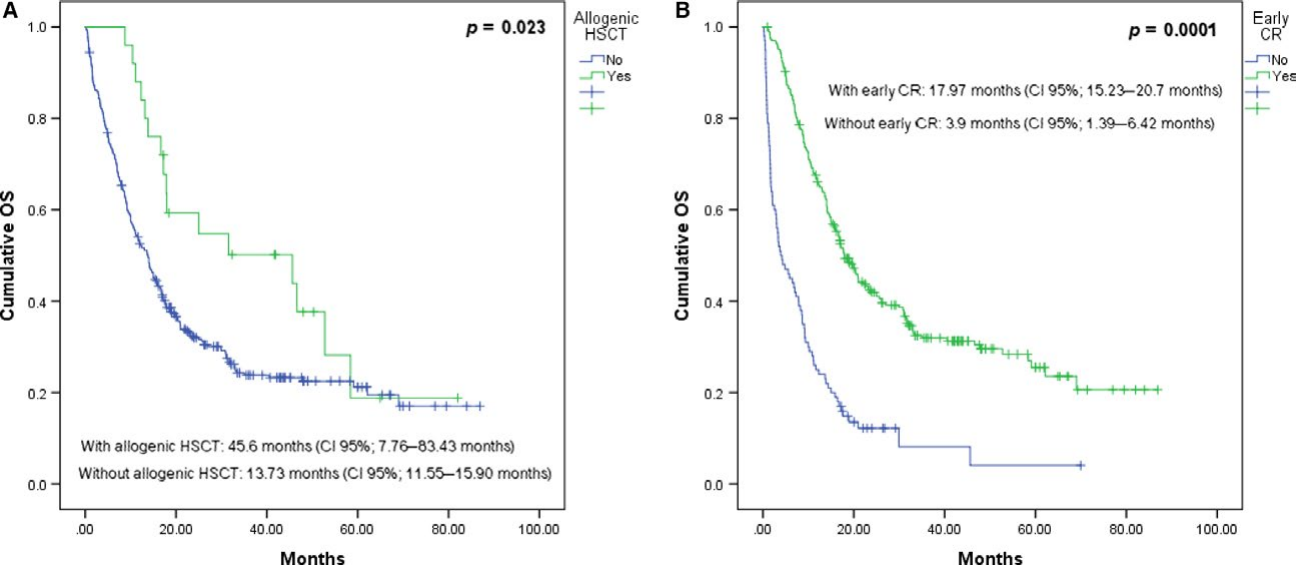
OS of patients with ALL in the AYA group based on whether or not they received an allogenic HSCT (1A) or achieved an early CR (1B).

At the time of the study, 410 patients had died (73.3%). The main causes of death were disease progression in 228 (55.6%), infection 110 (26.8%), hemorrhage 34 (8.2%), and chemotherapy‐induced toxicity 11 (2.6%). Multivariate analysis of the OS revealed the following risk factors: age group [HR: 1.292 (CI 95%: 1.099–1.520; *P *=* *0.002)], DFS [HR: 1.847 (CI 95%: 1.329–2.566; *P *=* *0.0001)], and LFT ≥2.5 ULN [HR: 1.604 (CI 95%: 1.198–2.148; *P *=* *0.002)]; the established protective factors were an early CR [HR: 0.309 (CI 95%: 0.246–0.388; *P *=* *0.0001)] and allogenic HSCT [HR: 0.469 (CI 95%: 0.297–0.739; *P *=* *0.001)]. Figure 1A and B show the effect of the protective factors in the AYA population.

### AYA group

**Figure 2:**
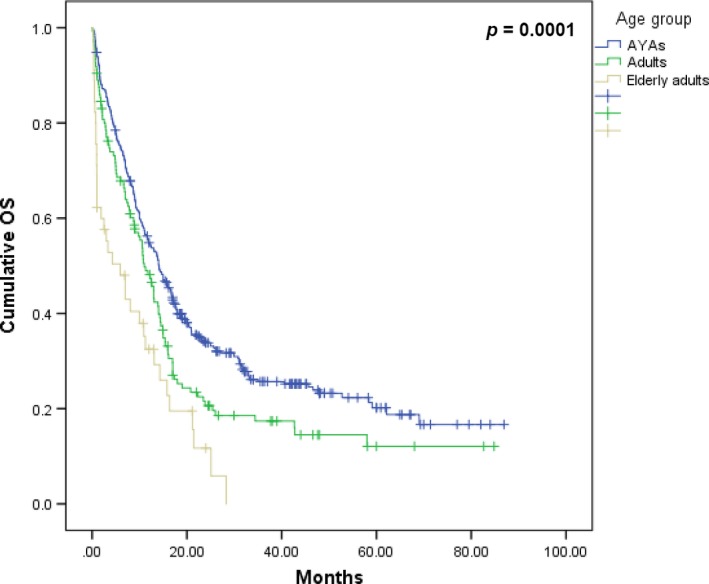
Comparison of OS by age group in the entire cohort.

A subanalysis of the AYA group was performed and we found that the only significant differences in their baseline data were the Ph^+^‐ALL cases, whose incidence was lower (Table 1). In terms of treatment response, the CR rate was 80%; however, the relapse rate was 61.7% (Table 2). As to the OS, the AYA group had a greater OS compared with the adults and elderly adults; this was statistically significant and the difference was 3.5 months: OS 14.12 months (CI 95%: 11.76–16.48 months) versus 10.58 months (CI 95%: 8.28–12.87 months; Fig. 2). Further, the AYA group had different risk factors than the adult and elderly adult groups (Tables 3 and 5).

**Table 3:** Cox multivariate analysis of OS in AYA patients

Variable	B coefficient	HR	CI 95%	*P* value
Risk factors				
TLS	0.509	1.663	1.124–2.462	0.011
LFT ≥2.5 UNL	0.490	1.663	1.147–2.325	0.007
Protective factors				
Early CR	−1.227	0.293	0.204–0.421	0.0001
Allogenic HSCT	−0.709	0.492	0.292–0.828	0.008
Platelets	−0.004	0.996	0.995–0.998	0.0001

**Figure 3:**
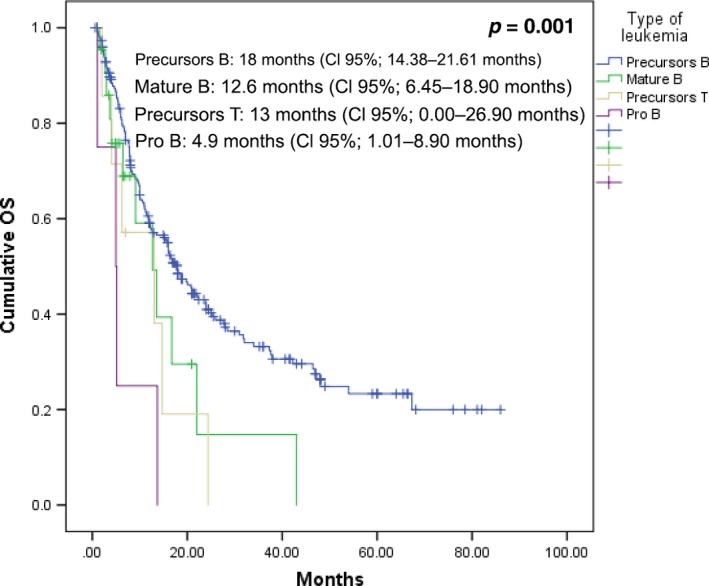
DFS of AYA patients that achieved CR, based on their leukemia sub‐type.

In the AYA group, DFS was 16.98 months (CI 95%: 13.69–20.27 months). On multivariate analysis, factors relating to DFS were the type of leukemia, hyperleukocytosis syndrome, and chemotherapy delays (see Table 4). We were surprised by the fact that the leukemia subtype was the strongest risk factor for DFS; in the Kaplan–Meier curves (Fig. 3), we can see that patients with the greatest survival had precursor B‐cell ALL while cases with a T, pro‐B and mature B immunophenotypes had a worse survival. We conducted a subanalysis that only included precursor B‐cell cases and univariate analysis revealed that the only risk factor for DFS was the presence of the hyperleukocytosis syndrome, HR 1.77 (CI 95%: 1.038–3.04; *P *=* *0.036); delayed chemotherapy only yielded a tendency with a *P* value = 0.064.

**Table 4:** Cox multivariate analysis of DFS in AYA patients that achieved CR

Variable	B coefficient	HR	CI 95%	*P* value
Risk factors				
Type of leukemia	0.393	1.481	1.207–1.818	0.0001
Hyperleukocytosis syndrome	0.540	1.715	1.022–2.880	0.041
Chemotherapy delays	0.363	1.438	1.004–2.059	0.048

### Adults and elderly adults group

In this cohort, we found an increased frequency of adults and elderly adults: Of the 183 cases (32%), 45 (8%) of which were elderly (≥60 years). As previously mentioned, this group's OS was 10.58 months, significantly lower than that in the AYA group, and their DFS was 13.40 months (CI 95%; 11.23–15.56 months); it was also significantly to that of the AYA group (Tables 5 and 6). The fact that 28% of these patients were Ph^+^‐ALL is relevant.

**Table 5:** Cox multivariate analysis of OS in adult and elderly patients

Variable	B coefficient	HR	CI 95%	*P* value
Risk factor				
Thrombosis	0.261	1.298	1.035–1.627	0.024
Protective factor				
Early CR	−1.425	0.240	0.156–0.371	0.0001

**Table 6:** Cox multivariate analysis of DFS in adult and elderly patients that achieved CR

Variable	B coefficient	HR	CI 95%	*P* value
Risk factors				
Chemotherapy delays	0.719	2.053	1.223–3.444	0.006
Hyperleukocytosis syndrome	0.674	1.962	1.075–3.578	0.028

## Discussion

This multicentric report represents the largest series in Mexico of adult ALL patients in which a survival analysis and risk identification were obtained. The sample size allows us to confidently establish the relevant clinical characteristics and some of the laboratory data. Furthermore, data collection allowed us to determine the principal areas of opportunity in the participating centers. Such is the case of caryotype testing that historically has been described as deficient in the past few years in Mexico 1, 21, 23; however, some investigators have recently reported an improvement in this caveat as a result of the routine implementation of FISH or molecular techniques to identify cases of Ph^+^‐ALL 22, 23, 25. Another multicentric study was recently conducted in Mexico, including six referral hematological centers, of a large series of patients with acute myeloid leukemia and ALL; however, they did not conduct a survival analysis nor risk factor identification. That study did show that the median age of patients with ALL was similar to that of our cohort: 28 years (14–81) versus 31 (16–88), respectively. Likewise, we found that gender distribution was also similar: males (53.8%)–females (46.2%) versus males (53.3%)–females (46.6%), respectively 29. It is striking that no research group to date has reported a frequency of Ph^+^‐ALL close to 25% as referred in developed societies 11; the overall frequency of Ph^+^‐ALL is estimated to be about 14% in Mexico. The group at the *Hospital Universitario de Monterrey* 25 found a small subgroup in their series (10/38 cases) with a Ph^+^ caryotype, 26.3%; however, in that same article, they report a frequency of 13.5% among the 74 cases analyzed by FISH. In our study, the incidence of Ph^+^‐ALL was 16.7% in the entire cohort, but of 28% in the adult and elderly adult group which is more in accordance with the previously mentioned data on its incidence in other populations 11. Still, we must remember that caryotype analysis or FISH were only obtained in 55% of cases so perhaps the Ph^+^‐ALL population remains underreported 30, 31, 32.

Another limitation of our study of a retrospective nature, is the scarce availability of certain immunophenotype markers such as aberrant myeloid markers; this precludes trustworthy correlation analyses between aberrant markers and the Philadelphia chromosome status, as well their inclusion in the survival analysis. ALL‐T cases were identified in 2.9%; which is less than previously reported by other authors in Mexico (5–10%) as well as in other countries (10–30%) 21, 22, 23, 24, 25, 29, 33, 34, 35, 36. We do not have an explanation for this finding.

Due to the lack of cytogenetic analysis in a large proportion of patients (45%), it is possible that we have subestimated high‐risk patients. However, with the available data we were able to document high‐risk disease in at least 52% of cases. In the studies conducted in Mexico, the frequency of high‐risk patients is estimated to be between 59% and 81%, but criteria have changed over time 1, 23, 24, 25, 37. In American and European developed societies, the risk classification has also fluctuated between 47% and 77%, due to modifications in criteria over time 26, 38.

We observed a great diversity of treatment options in our study, encompassing known and previously published chemotherapy regimens such as hyper‐CVAD and BFM 26, 27, as well as other locally designed and protocols based on pediatric experience. It is also possible that the hyper‐CVAD regimen was applied in different ways in different institutions. Any variability precludes us from comparing treatments and since we found that apparently, treatment is not a significant variable in survival analysis, it should be interpreted cautiously due to the previously mentioned factors as well as to other possible confusers.

The most frequently used treatment protocol was hyper‐CVAD, but the CR and relapse rates as well as the survival curves were inferior to those reported by developed societies 13, 14, 15, 16 and this has been a constant feature in the country's publications 21, 24. Although we found that the AYA group had better CR rates (80.1%) in comparison with the adult and elderly adult group (65.6%), these rates are still low compared with the data reported by developed societies 13, 14, 15, 16. This may result from the toxicity attributable to the hyper‐CVAD regimen in our population which is characterized by particular socioeconomic features, as well as to treatment initiation delays (20% of cases in our cohort), unlike the data reported by MDACC in which the median interval between chemotherapy applications was 20 days 33; Mexico City investigators have also demonstrated the negative impact of chemotherapy delays on DFS 39, 40.

Regimens inspired by pediatrics, particularly the addition of l‐asparaginase to the treatment protocols in the AYA group do appear to improve long‐term survival: 60% versus 26–46% with adult regimens 34, 41, 42, 43. Unfortunately, most patients in our cohort were not treated with this drug and it was administered to only 10.2% of the patients in the AYA group, which could be a related factor in their low survival rates. Two previous reports in Mexico of ALL patients treated with l‐asparaginase‐containing protocols showed a 5‐year OS close to 50%, which supports the inclusion of this drug in the treatment of patients under 40 years of age 44, 45.

The administration of rituximab has been proven to improve survival in CD20^+^ patients 3, but we were unable to estimate how many patients received rituximab in this study. This concurs with the observation in our cohort whereby OS was lower in ALL cases with a mature‐B‐cell phenotype. The fact that these patients must be treated with specific regimens and that they benefit from adding rituximab to hyper‐CVAD has been previously demonstrated 46, 47. We also found they had a decreased survival which may be explained by the lack of rituximab administration or their regimen was not designed for their leukemia subtype. However, this must be cautiously interpreted since these features were observed in approximately 50% of cases, or surface immunoglobulin was not detected by immunophenotyping.

As we previously mentioned, only 68% of the Ph^+^‐ALL patients (3–5% of the entire cohort) received a TKI (imatinib or dasatinib). This may have contributed to the high incidence of relapse and the short OS of our population.

Another important aspect to discuss is the incidence of treatment‐associated mortality; there is a significant percentage of deaths unrelated to relapse (21.2%) while the expected rate using adult protocols should be <15% and <5% with pediatric‐style regimens 26, 39, 48, 49.

We identified in our study 52.1% high‐risk patients and only 5.7% of our cohort received an allogenic HSCT; this is also a limitation in the participating centers since allogenic HSCT improve survival when offered to young adults 35, 36. All the authors of the present study agreed that many of these high‐risk patients could have benefited from this procedure. We cannot identify the precise causes of this finding among the participant institutions, but this demonstrates the need to include the HSCT into the treatment strategies.

Some survival risk factors such as the leukocyte count and age have been previously described by many authors 11, 14, 17, 20, 21, 26. We were surprised by the fact that LFT ≥2.5 UNL is an independent risk factor for survival; this has been previously described in studies of acute myeloid leukemia patients in Mexico 50. We believe that this finding may be due to a combination of several factors in our population, such as advanced disease with liver infiltration, clinical deterioration, a high prevalence of fatty liver and obesity. We must emphasize that the prevalence of obesity in our report is high (18.5%) although there are missing data on patient body mass index in our overall cohort.

The negative impact of the Ph‐like signature has been recently described, as well as its prevalence in the Latino population 32, 51. We do not know the frequency of this feature in our study population but a high prevalence is to be expected in the light of previous reports and it tends to negatively affect survival.

The authors of this study, as a group, believe that the great variability in our population's strata and socioeconomic conditions, negatively compromises the survival of the adult population with ALL, but such an analysis is beyond the scope of this study.

## Conclusions

This study represents the largest multicentric series of adult patients with ALL in Mexico in which a survival analysis is performed. The particularities of this patient population warrant the development of prospective studies in our country so as to standardize treatment regimens on the basis of the patients’ age group and particularly in the AYA group, representing most of our patients and that require pediatrically based regimens including l‐asparaginase, TKIs in Ph^+^‐ALL patients, and that may increase CR rates and survival curves. It is also important to improve standards of care in order to decrease treatment‐related mortality. It is necessary to improve diagnostic technological aspects in the participating institutions, such as cytogenetics, FISH and molecular testing to identify subgroups, including those with the Ph‐like signature; the number of patients admitted into HSCT protocols must also be increased. All of these discussed aspects must be seriously taken into account by Federal Health Care Programs (*Seguro Popular*).

Despite its limitations, we believe that to date, this study reflects the most solid reference in terms of survival analysis of adult ALL patients in Mexico; in view of the many socioeconomic similarities, it could also be an important reference throughout Latin America.

## Conflict of Interest

None declared.
